# Supplementary material for: Novel Driver Strength Index highlights important cancer genes in TCGA PanCanAtlas patients
Source: PeerJ. 2022 Aug 11;10:e13860. doi: 10.7717/peerj.13860 (PMC9375969; doi:10.7717/peerj.13860)
Supplement: Supplemental Information 2 [file peerj-10-13860-s002.zip › COHORTS/genes plots/cohorts/2021_8_16_14_9_distribution_cohorts_DLBC.pdf]

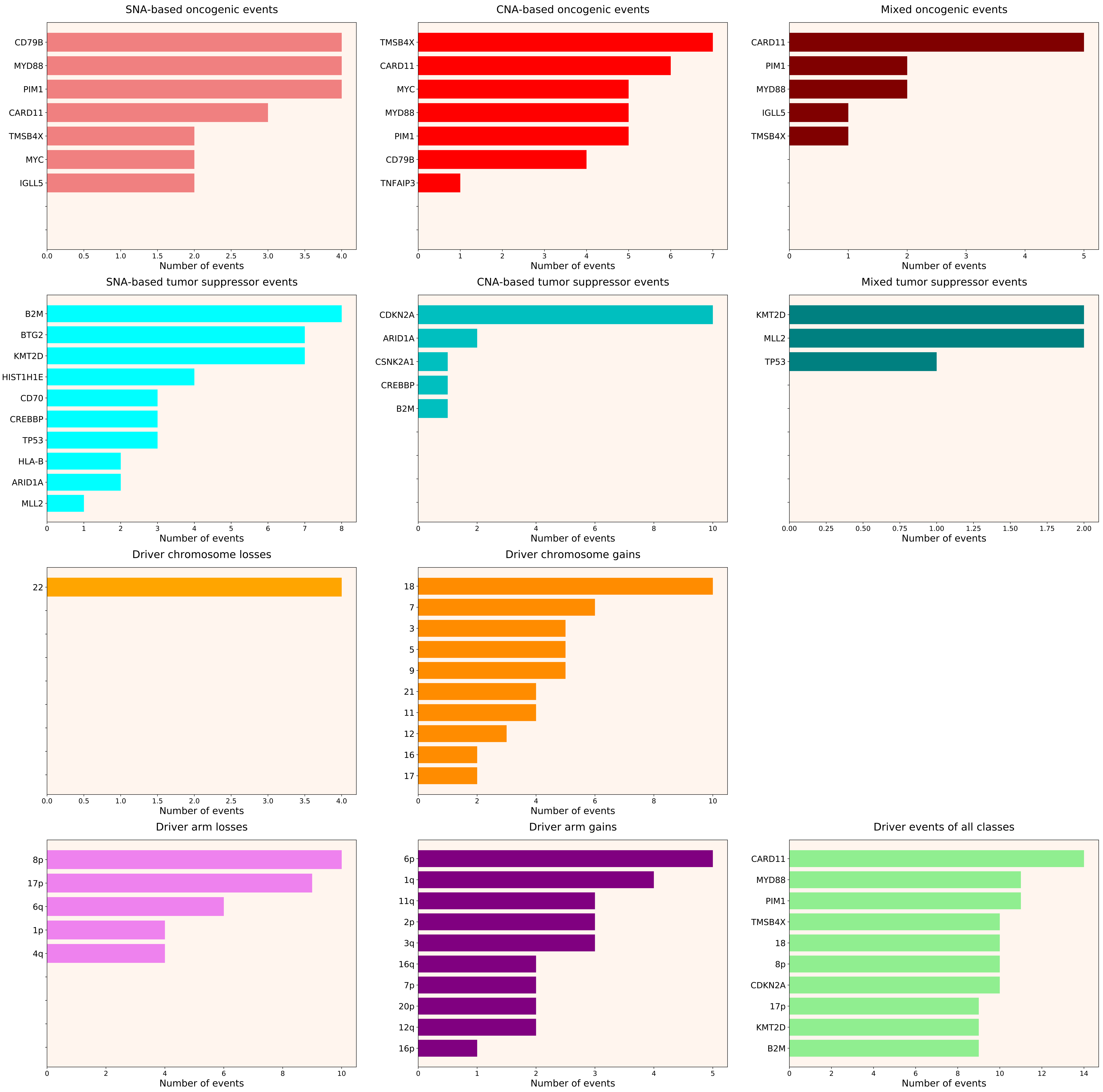

SNA-based oncogenic events
CD79B
MYD88
PIM1
CARD11
TMSB4X
MYC
IGLL5
Number of events
CNA-based oncogenic events
TMSB4X
CARD11
MYC
MYD88
PIM1
CD79B
TNFAIP3
Number of events
Mixed oncogenic events
CARD11
PIM1
MYD88
IGLL5
TMSB4X
Number of events
SNA-based tumor suppressor events
B2M
BTG2
KMT2D
HIST1H1E
CD70
CREBBP
TP53
HLA-B
ARID1A
MLL2
Number of events
CNA-based tumor suppressor events
CDKN2A
ARID1A
CSNK2A1
CREBBP
B2M
Number of events
Mixed tumor suppressor events
KMT2D
MLL2
TP53
Number of events
Driver chromosome losses
22
Number of events
Driver chromosome gains
18
7
3
5
9
21
11
12
16
17
Number of events
Driver arm losses
8p
17p
6q
1p
4q
Number of events
Driver arm gains
6p
1q
11q
2p
3q
16q
7p
20p
12q
16p
Number of events
Driver events of all classes
CARD11
MYD88
PIM1
TMSB4X
18
8p
CDKN2A
17p
KMT2D
B2M
Number of events
